# The effects of education based on extended health belief model in type 2 diabetic patients: a randomized controlled trial

**DOI:** 10.1186/2251-6581-12-45

**Published:** 2013-10-28

**Authors:** Fatemeh Bayat, Davoud Shojaeezadeh, Masoud Baikpour, Ramin Heshmat, Maryam Baikpour, Mostafa Hosseini

**Affiliations:** 1 Department of Health Education and Promotion, School of Public Health and Institute of Public Health Research, Tehran University of Medical Sciences, Tehran, Iran; 2 School of Medicine, Tehran University of Medical Sciences, Tehran, Iran; 3 Department of Endocrinology, Endocrinology and Metabolism Research Center, Tehran University of Medical Sciences, Tehran, Iran; 4 Department of Epidemiology, Chronic Diseases Research Center, Tehran University of Medical Sciences, Tehran, Iran; 5 School of Medicine, Shahid Beheshti University of Medical Sciences, Tehran, Iran; 6 Department of Epidemiology and Biostatistics, School of Public Health and Institute of Public Health Research, Tehran University of Medical Sciences, Tehran, Iran

**Keywords:** Educational intervention, Extended health belief model, Type 2 diabetes

## Abstract

**Background:**

Type II diabetes and its complications impose a large economic burden on health care systems. This study aims to assess the effectiveness of educational intervention based on extended health belief model on type 2 diabetic patients.

**Methods:**

120 patients with type II diabetes referring to randomly selected hospitals of Tehran University of Medical Sciences were enrolled in this educational intervention study. Patients were randomly divided into two groups (intervention and control). Data were collected using a questionnaire including demographic information and extended health belief model constructs. Two face to face educational sessions were conducted for each patient. Data were collected in two groups at three stages of the study; before the educational sessions and at 3 months and 6 months intervals. Analysis was performed by SPSS (17.0) and STATA (11.0) using independent T-test, Chi-square, Fisher’s exact test, analysis of covariance and Generalized Estimating Equation. A p value of less than 0.05 was regarded as statistically significant.

**Results:**

The educational program had a positive and significant impact (p < 0.0001) on extended health model belief constructs (including perceived susceptibility, perceived intensity, perceived benefits, perceived barriers and self-efficacy) in experimental group, 3 and 6 months after the intervention.

**Conclusions:**

The results of this study showed the importance of extended health belief model based education in improving the model constructs and increasing self-efficacy in patients with type-2 diabetes.

## Introduction

Diabetes mellitus is a major and growing health problem affecting more than 180 million people worldwide and according to the estimates the number is expected to double by 2030 [1]. As a result of population ageing and increasing prevalence of obesity, type 2 diabetes accounts for most of the cases. Diabetes complications are very common and impose a large economic burden on the patient and society [2]. In the short term, type-2 diabetes may lead to symptoms and debilities and in the long term to more serious complications [3]. Unhealthy behavior such as lack of physical activity, consumption of high-fat and high-calorie foods, incorrect blood glucose measurement and inadequate attention to medication regimens are known risk factors for complications’ aggravation. Type 2 diabetes and its chronic complications impose a substantial burden on health care systems. It is common in Middle East; it has a prevalence of 29% in United Arab Emirates [4], 7.7% in Iran [5] and a prevalence of 14% in Tehran. One out of five people in Iran over the age of 30 is diagnosed with diabetes and overall it has become a global epidemic [6].

Studies show that those theory-based educational programs that apply cognitive frameworks can have a positive effect on the results. A few of these programs are currently part of the primary care, but they have not yet been specifically used to educate diabetic patients [3, 7].

Extended health belief model (EHBM), the theoretical framework applied in this study, is used to assess the patients’ motivation to adapt to a health related behavior. This model consists of six key constructs affecting health behaviors including perceived susceptibility, perceived severity, perceived benefits, perceived barriers, cues to action and self-efficacy [8].

Perceived susceptibility is a person’s opinion regarding his or her personal chances of developing a condition. An individual’s opinion about the seriousness of a specific condition and its consequences is referred to as perceived severity. “Perceived benefits” refers to the patient’s belief in the efficacy of the advised action to reduce risk or seriousness of impact. Any impediments in the way of adopting a recommended health-related behavior, is considered as the individual’s perceived barrier. Self-efficacy is a newly added construct that lies at the center of Bandura’s social cognitive theory. Influenced by modulator variables, self efficacy is now a key component proposed in most of the recent models [9] and it was added to the original four beliefs of HBM in 1998. Bandura defines perceived self-efficacy as an individual’s judgment of his or her capabilities to organize and execute courses of action required to attain designated types of performances [10].

To date there have been few studies conducted on the effects of HBM-based educational intervention [8, 11] and non-HBM-based ones [7, 12–14] on diabetic patients. But this study aimed to assess the impact of EHBM based education on patients with type 2 diabetes. The difference between this study and other recently conducted studies is the addition of the self-efficacy construct to the variables and adding follow ups with 3 and 6 month intervals which gives us a better appreciation of the long term effects of educational intervention on the model’s constructs.

## Methods

This study is an educational intervention based on Extended Health Belief Model (EHBM), conducted on type 2 diabetes patients referring to randomly selected hospitals of Tehran University of Medical Sciences (Dr Shariati, Baharlou and Amir Alam hospitals), in 2012.

The sample-size for both experimental and control groups was calculated using the following formula with a confidence interval of 95% (α = 0.05) and statistical power of 80% (β = 0.20).

$$n=\frac{{\left(Z_{1-\alpha /2}+Z_{1-\beta }\right)}^{2}\times \left[p_{1}\times \left(1-p_{1}\right)+p_{2}\times \left(1-p_{2}\right)\right]}{{\left(p_{1}-p_{2}\right)}^{2}}$$

Based on this formula, each group was calculated to have a sample size of 55 patients. Taking into account a 10% probable loss of samples during the study, the calculated sample size was increased to 60 patients for each group. Patients were randomized to two groups using a permuted block size of 4. Inclusion criteria were predefined as follows: being able to read and write, and diagnosis of the type 2 diabetes confirmed by a specialist. Exclusion criterion: Patients unwilling to participate in the study. A structured questionnaire was used as a data collection tool, including three sections regarding basic demographic data (19 questions), Health Belief Model constructs (19 questions) and self-efficacy (8 questions). HBM constructs were measured using a five point Likert scale ranging from 1 (strongly disagree) to 5 (strongly agree). As for the perceived barriers, the scaling was opposite the other constructs. Scoring the self-efficacy section of the questionnaire was based on the patient’s ability to adopt a specific behavior ranging from 1 to 10.

Participants in each group completed an interview-based questionnaire, the results of which were analyzed to design an EHBM-based educational content for the experimental group.

The content included:

● Information about diabetes, its risk factors, complications and treatments.

● Education on exercise programs in patients with type-2 diabetes including the type, duration, frequency and intensity of exercise.

● Checking pre-exercise blood glucose level and measures that should be taken in case of hypoglycemia during exercise.

● Education about diet including the type and amount of food according to the diabetes food pyramid and the timing of consumption.

● Perceived susceptibility and intensity including the statistics on this subject, the causes of diabetes complications, application of motivating methods, explaining the risks of disregarding the diet, medical regimen and exercise program, encouraging the patients to stick to their therapeutic regimen to avoid the complications, emphasizing the controllable nature of diabetes and introducing the methods.

● The benefits and obstacles of nutritional diet and exercise program and proposing methods to increase the patient’s knowledge about these subjects.

● Methods to decrease perceived barriers, recommending patients to join organizations such as diabetes association, information on how to reduce the expenses related to the diabetic nutrition, educating the patients who had amputations about exercises to strengthen each limb, methods to control and prevent the complications.

The educational program consisted of two 30 to 45 minute sessions, presented via pamphlets and face to face lectures using the “question and answer” method. The “question and answer” method was used to encourage the participants to get involved in the process and to make sure that they were paying attention. Also follow-up phone calls were made to the participants after the program.

Prior to enrolling the participants in the study, informed consent was obtained from each person based on the statutes passed by the ethics committee of Endocrinology and metabolism Research Center (EMRC) of Tehran University of Medical Sciences, and the principles of the Declaration of Helsinki were applied throughout the study.

The content validity of the questionnaire was evaluated based on the feedbacks of research experts in this field. The test-retest method was used to estimate the questionnaire’s stability and consistency. The questionnaire was completed twice by 15 type 2 diabetic patients with a 10-day interval and data were recorded in two phases, before and after training. Analysis showed no significant difference in the responses to 19 questions of HBM (84%) and 16 questions about self-efficacy (87.5%). This indicates a stability of 84%.

The contents of the educational program included: definition of diabetes, signs and symptoms, the importance of control and prevention of short and long term complications, blood glucose level measurement methods, diet control methods and physical activity. Post-test questionnaires were completed by both experimental and control groups 3 and 6 months after the intervention. Analysis was performed by SPSS (17.0) using Chi-square and Fisher’s exact test for demographic variables, and independent T-test along with analysis of covariance (ANCOVA) for the HBM constructs and self-efficacy data. Also, to estimate the trend of intervention effect Generalized Estimating Equation (GEE) were applied using STATA (11.0). A p value of less than 0.05 was regarded as statistically significant.

The importance of the subject and its objectives were clearly explained to the patients, to encourage their active participation in the study, and they were reassured that their information will remain confidential.

## Results

Table 1 shows the basic demographic characteristics of the patients. Most of the patients in both groups were women, married and aged between 36 and 55. The Chi-square and Fisher’s exact test showed no significant difference between neither the distribution of these variables and education, nor the duration of diabetes and the duration of diabetic association membership (p > 0.05).

**Table 1:** **Distribution of demographic characteristics of patients according to intervention**

Demographic characteristics		Cases		Controls		P-value
		No	Percent	No	Percent	
Age	35-45 yr	22	36.7%	22	36.7%	0.56^a^
	36-55 yr	25	41.7%	29	48.3%	
	56-65 yr	13	21.7%	9	15.0%	
Gender	Female	34	58.6%	31	51.7%	0.45^a^
	Male	24	41.4%	29	48.3%	
Marital status	Single	6	10.0%	4	6.7%	0.51^a^
	Married	54	90.0%	56	93.3%	
Education	Elementary	19	31.7%	17	28.3%	0.88^**a**^
	Guidance	17	28.3%	21	35.0%	
	High school & diploma	17	28.3%	15	25.0%	
	University	7	11.7%	7	11.7%	
Member of diabetes society	Yes	17	28.3%	16	26.7%	0.84^a^
	No	43	71.7%	44	73.3%	
Disease history	1 years	10	16.7%	11	18.3%	0.74^b^
	2-5 years	37	61.7%	38	63.3%	
	6-9 years	11	18.3%	11	18.3%	
	>9 years	2	3.3%	0	0.0%	

a) Chi-squared test.

b) Fisher Exact test.

The mean scores of the HBM constructs before the intervention were similar in both experimental and control groups and there were no significant differences (p > 0.05, Table 2). But the mean score of perceived susceptibility construct increased from 15.85 (±1.6) before the intervention, to 17.77 (±2.1) 3 months after that, which was calculated to be statistically significant using the analysis of covariance (p < 0.0001). The mean score at the 6 month interval after the intervention was 17.78 (±2.2) (Table 2). Although it was still statistically significant compared to the baseline (p < 0.0001, Table 2), the susceptibility score remained constant over 3 to 6 month (p = 0.29).

**Table 2:** **Mean (±SD) of components of health belief model before and after intervention**

Components	Group	Before intervention	P^a^	After 3 month	P^b^	After 6 month	P^c^	P^d^
		(Mean ± SD)		(Mean ± SD)		(Mean ± SD)		
Susceptibility	Case	15.85 ± 1.6	0.33	17.77 ± 2.1	<0.0001	17.78 ± 2.2	<0.0001	0.29
	Control	15.82 ± 1.3		15.95 ± 1.4		15.95 ± 1.4		
Intensity	Case	21.48 ± 1.3	0.88	23.58 ± 1.3	<0.0001	24.12 ± 0.9	<0.001	<0.001
	Control	21.45 ± 1.3		21.60 ± 1.2		21.62 ± 1.4		
Benefits	Case	17.62 ± 1.6	0.61	18.93 ± 1.2	<0.0001	19.18 ± 0.9	<0.001	<0.001
	Control	16.68 ± 1.8		16.93 ± 1.7		16.77 ± 1.8		
Barriers	Case	14.90 ± 2.6	0.86	12.0 ± 3.6	<0.0001	11.55 ± 3.4	<0.001	<0.001
	Control	14.82 ± 2.8		14.48 ± 3.0		14.68 ± 3.1		
Self-efficacy	Case	22.88 ± 2.8	0.68	44.38 ± 4.3	0.0001	44.40 ± 4.3	<0.001	0.11
	Control	23.08 ± 2.8		23.20 ± 2.4		23.27 ± 2.4		

a) Independent sample T-test at baseline; b) ANCOVA after 3 months adjusting for baseline, c) Independent sample T-test after 6 months; d) ANCOVA after 6 months adjusting for 3 months.

The mean score of perceived intensity before the intervention was 21.48 (±1.3) in the experimental group. It increased to 23.58 (±1.3) (p < 0.0001) after 3 months, and in 6 months it reached 24.12 (±0.9) (p < 0.00001), which was also found to be statistically significant (Table 2).

The mean score of perceived barriers before the educational sessions was 14.90 (±2.6) which decreased to 12 (±3.67) (p < 0.0001) after 3 months and reached 11.55 (±3.4) (p < 0.001) at the 6 month interval. Both these changes were statistically significant (Table 2). Contrarily the mean score of self-efficacy increased over time, from 22.88 (±2.8) before the intervention to 44.38 (±4.3), 3 months after that, and in 6 months it reached 44.40 (±4.3) (p < 0.0001, Table 2).

It is worth mentioning that the main assumptions of ANCOVA were checked for various components of EHBM and found reasonable. Also, as in this study the total sample was 120, according to Central Limit Theorem applying the regression procedure (ANCOVA) which is a parametric tool is fine.

Besides, as the follow ups were at 3 and 6 month intervals, to estimate the trend of intervention effect we used GEE procedure in STATA (11.0) taking account of the dependency of measurements and found that the monthly trend of intervention effect for the model constructs: susceptibility, intensity, benefits, barriers, and self-efficacy were 0.35 (0.28-0.41), 0.37 (0.32-0.42), 0.33 (0.27-0.38), 0.38 (0.28-0.48) and 3.92 (3.58-4.25), respectively.

## Discussion

Education is the cornerstone of diabetes management, so finding a suitable method to improve self-efficacy is of great importance. Since there have been only a few HBM-based studies on self-efficacy in diabetic patients, this study was conducted to examine the effect of Extended Health Belief Model on the self-efficacy of patients with type 2 diabetes.

In this study 25 patients from the experimental group (41.7%) and 39 patients from the control group (48.3%) belonged to the age group of 36–55 years. As for the marital status, 54 patients (90%) from the experimental group and 56 patients (56.9%) from the control group were married. At the starting point of the study there were no significant differences between the distribution of demographic variables in both case and control groups (p > 0.05).

The results of this study showed an increase in the mean scores of perceived susceptibility, perceived severity, perceived benefits and self-efficacy, and also a decrease in the mean score of perceived barriers after the implementation of the educational program on the experimental group.

As shown by the results of this study participating in the educational program increased the mean score of perceived susceptibility in the experimental group in both 3 and 6 month intervals (p < 0.0001).

Baghianimoghadam et al. (2010) conducted a study assessing the effects of current education compared to peer-education on walking in type 2 diabetic patients, based on Health Belief Model. Their results showed a significant increase in the mean scores of perceived susceptibility and perceived severity in the case group after educational intervention, which matched our findings [15].

The mean scores of perceived severity and perceived benefits in the experimental group increased 3 and 6 months after the educational intervention (p < 0.0001). The results of Shamsi et al’s study conducted on 88 patients with type 2 diabetes also showed an increase in the mean scores of perceived susceptibility, perceived severity and perceived benefits, and a decrease in perceived barriers, 3 months after the intervention [16].

Compatible with the results of this study, Mardani et al. also carried out an educational program for patients, that increased the mean score of the benefits construct and led to a decrease in the mean score of the perceived barriers construct [17]. A similar study by Sharifirad et al. verified these findings [18].

Aghamolai et al. conducted an experimental research and examined the effects of Heath Belief Model application on modifying the self-care behaviors in type 2 diabetic patients. The results showed that after the educational intervention a significant increase occurred in all the constructs of the model and the perceived barriers construct decreased significantly [19]. The same results were obtained from another interventional study performed by Sharifirad et al. (2008) on 88 patients with type-2 diabetes [20].

At the beginning of the study, the mean score of self efficacy was 22.88 (±2.85), which increased to 44.38 (±4.35) after 3 months and reached 44.40 (±4.31) in the 6 months follow-up (p < 0.0001). It indicated that the increasing trend was constant (p = 0.11). In 2011 in Patrick’s study, aimed to assess the effect of diabetic patient’s education and self-management education in type-2 diabetes, the results showed a significant increase in the mean score of self-efficacy 6 months after the educational intervention, which is compatible with the results of this study [21].

Heijden et al. in 2012, testing the effects of an exercise intervention, based on self-efficacy for inactive patients with type-2 diabetes, showed a significant improvement in the experimental group’s self-efficacy [22]. Baljani et al. also showed a significant increase in every aspect of the self-efficacy construct, examining the effect of education on promoting self-efficacy on patients with cardiovascular disease [23]. Proving the same point was the study performed in 2008 by Atak et al. determining the effect of education on knowledge, self management behaviors and self-efficacy of patients with type-2 diabetes [24].

One of the limitations in conducting this study was not having a suitable place to hold the educational sessions, which led to choosing the more effective, face to face education method. Following the patients 3 and 6 months after the intervention was another problem. Other obstacles include using a report-based data collecting tool, inaccessibility of the patient’s medical records for the physician to assess patient’s improvement, and our inability to generalize the results to the majority of the population.
